# Neutropenia, agranulocytosis and dipyrone

**DOI:** 10.1590/S1516-31802005000500009

**Published:** 2005-09-01

**Authors:** Nelson Hamerschlak, Alexandre Biasi Cavalcanti

**Keywords:** Agranulocytosis, Neutropenia, Dipyrone, Incidence, Case-control studies, Agranulocitose, Neutropenia, Dipirona, Incidência, Estudos de casos e controles

## Abstract

**CONTEXT:**

Neutropenia and agranulocytosis may be defined as granulocyte counts of less than 1,500/mm^3^ and 500/mm^3^, respectively. Agranulocytosis is a rare and serious disease often caused by drugs. Its mortality rate is around 10%. The most common manifestations are infections such as tonsillitis, pharyngitis, stomatitis or pneumonia. Although dipyrone is one of the drugs known to be associated with agranulocytosis, the strength of the association has been a matter of much debate. Moreover, alternative analgesic and antipyretic agents are not devoid of serious side effects.

**CONCLUSIONS:**

It is therefore necessary to establish the incidence of agranulocytosis in Latin America and the role of dipyrone. The ongoing LATIN Study is a multicenter international case-control study that will provide answers for these questions.

## RESUMO

**CONTEXTO:**

Neutropenia e agranulocitose podem ser definidas como contagem de granulócitos inferior a 1.500/mm^3^ e 500/mm^3^, respectivamente. Agranulocitose é uma doença séria e rara, freqüentemente causada por medicações. A letalidade é de aproximadamente 10%. As manifestações clínicas mais comuns são infecções como tonsilite, faringite, estomatite e pneumonia. A dipirona é uma das medicações sabidamente associadas a agranulocitose. Entretanto, a intensidade desta associação tem sido motivo de muita controvérsia. Adicionalmente, analgésicos e antipiréticos alternativos não são livres de efeitos adversos graves.

**CONCLUSÕES:**

Desta forma, é necessário estabelecer a incidência da agranulocitose na América Latina e o papel da dipirona. Com o objetivo de responder estas questões, está em andamento o LATIN, um estudo caso-controle, multicêntrico, internacional.

## INTRODUCTION

Neutropenia may be defined as a neutrophil granulocyte count of less than 1,500/mm^3^. This definition can be applied to any age group, although certain groups may be interpreted in relation to some particular characteristics. For instance, blacks and Yemeni Jews usually present lower granulocyte count. Agranulocytosis may be defined as a neutrophil count of less than 500/mm^3^.

The peripheral neutrophil count reflects the balance between several compartments. In the bone marrow, there is the mitotic pool, the maturation pool and the storage pool. Outside the bone marrow, there are circulating pools, the marginated pool adhering to the vascular endothelium and the tissue pool. Clinical trials to establish the number of neutrophils measure only the circulating pool in transit from the bone marrow to tissues. The movement is usually made in the direction bone marrow-blood-tissue.

The biology of hematopoiesis is complex and regulated by many cytokines. Some cytokines, such as G-CSF and GM-CSF (granulocyte colony-stimulating factor and granulocyte-monocyte colony-stimulating factor), together with complementary components, are able to increase the release of granulocytes from the bone marrow storage pool to circulation. This may result in a two to threefold increase in the number of leukocytes within four to five hours. The marginated pool, representing over half the number of granulocytes in peripheral blood, may also be released to circulation. Epinephrine is one of the main elements in this process, which explains the neutrophilic response to stress and exercises. Many other mediators are involved, such as L-selectin, P-selectin, lactoferrin and acid isoferritins.

Out of a total of 1.2 x 10^9^ granulocytes/kg, 20% are precursors of the bone marrow pool, 75% are in the storage pool, 3% in the marginated pool and 2% in the circulating pool. Under normal conditions, 1.5 x 10^9^ granulocytes/kg are produced per day. Inflammatory processes increase this production. The granulocytes live for nine days in the bone marrow, three to six hours in blood and one to four days in tissues. Therefore, it must be borne in mind that any interpretation of results should consider that measurements in the peripheral blood represent a small share of neutrophils and a short period of useful life.

Neutropenia may occur because of decreased production of granulocytes, shift of granulocytes from the circulating compartment to the marginated or tissue pool, peripheral destruction, or a combination of these three mechanisms (Table 1).^1^ Many agents can cause neutropenia. The mechanism may directly involve the bone marrow, such as in the case of antineoplastic agents; be related to formation of antibodies and complements against hematopoietic precursors; and, more rarely, involve peripheral destruction with neutrophil clearance.

**Table 1.** Causes of neutropenia

Acquired neutropenia
Post-infectious
Drug-induced
Benign familial neutropenia
Benign chronic neutropenia in children
Chronic idiopathic neutropenia
Autoimmune neutropenia
Isoimmune neutropenia
Neutropenia associated with metabolic disorders
Neutropenia due to excessive margination
Nutritional deficiencies
**Intrinsic defects**
Kostmann syndrome
(severe congenital neutropenia)
Myelokathexis/neutropenia with tetraploid nuclei
Cyclic neutropenia
Shwachman-Diamond-Oski syndrome
Chediak-Higashi syndrome
Reticular dysgenesis
Dyskeratosis congenital

The main clinical manifestations of agranulocytosis are fever, tonsillitis, pharyngitis, sepsis, stomatitis and pneumonia. As most physicians know, it is a rare event and its incidence ranges from 1.7 to 7.0 cases/million individuals/year.^2^ The mortality rate is 10%, and complete recovery is expected in over 80% of patients.^3^ With appropriate antibiotic therapy and bone marrow growth factors, today this rate is tending to drop significantly.

### Incidence and etiology of agranulocytosis – the LATIN Study

The LATIN Study started in Brazil and has been extended to other Latin American countries, since there were no appropriate data on the incidence and risk factors of agranulo-cytosis and aplastic anemia in these countries. Data restricted to Brazil on the incidence and risk factors of aplastic anemia were presented by Maluf et al.^4^

The only data available on the incidence of agranulocytosis in Brazil are from a study published in 1993. This is a retrospective evaluation of some cases of drug-induced agranulocytosis in São Paulo, a city with 12 million inhabitants, and it showed an incidence of 0.44-0.82 cases/million inhabitants/year.^5^

The doubt about the use of medicines in cases of agranulocytosis is related to knowing which drug caused the problem. The patients presenting this disease have usually taken more than one drug. Which drug is involved: the one the patient took yesterday or another taken seven days ago? In the case of analgesic drugs or antipyretics, which came first, the chicken or the egg? Did the patient take an antipyretic because he/she had agranulocytosis or did the drug caused the problem?

The LATIN Study was designed to attempt to answer some questions about aplastic anemia and agranulocytosis by using a case-control study with evaluation of incidence. The primary objective was to assess the possible association of drugs, diseases and environmental and occupational factors with agranulocytosis and aplastic anemia. The secondary objectives were to estimate the incidence of agranulocytosis and aplastic anemia in Latin America and to assess the possible regional differences in the association of aplastic anemia and agranulocytosis with the risk factors mentioned.^6^

The eligibility criteria for agranulocytosis were defined as granulocytes < 500/mm^3^ with decreased granulocytic series in the marrow, or recovery of granulocyte count to normal values after 30 days; hemoglobin ≥ 10 g/dl, platelets ≥ 100,000/mm^3^, and bone marrow study (when performed) ruling out other diagnoses.^6^

For case-control studies, the appropriate choice of controls is as important as the selection of cases. Therefore, the eligible controls had to have:

- Been admitted within a three-month interval, at most, and to the same hospital or treatment center as the cases;
- Presented acute diseases, such as trauma, Presented acute diseases, such as trauma, appendicitis or acute infections. Alternatively, patients admitted for surgical treatment of oligosymptomatic conditions, such as cataract, hallux valgus, nasal septum deviation and plastic surgery, were accepted;
- Had any clinical conditions due to chronic ad any clinical conditions due to chronic disease complications ruled out;
- Been pair-matched for sex and age.

The controls were excluded from the study in the event of present or past histories of the following:

- Use of chemotherapy, radiotherapy and Use of chemotherapy, radiotherapy and immunosuppressants;
- Hematological and neoplastic diseases;
- Systemic diseases that are known to cause Systemic diseases that are known to cause neutropenia or pancytopenia, such as lupus, HIV infection and hypersplenism;
- Inability to answer the questionnaire.

The final sample will include 164 cases of agranulocytosis and 164 cases of aplastic anemia. Four controls will be enrolled for each case. A total of 1,640 patients are expected to be enrolled.

A trained investigator will interview the cases and controls by means of a standardized questionnaire. The information gathered by means of the printed Case Report Form (CRF) will be transferred to the database through an electronic data retrieval system via internet. This system has several mechanisms to help in gathering complete data and reducing inconsistencies.

The pilot study has already shown interesting results that were used for adjusting the definite protocol. Seven centers took part in the pilot study, representing the main regions in Brazil.^6^ Seventy-four cases of aplastic anemia, 16 of agranulocytosis and 103 controls were enrolled. Although no definite conclusion may be drawn, since this was not the purpose of the pilot study, the incidence was 0.5 cases/million individuals/year and 2.7 cases/million individuals/year for agranulocytosis and aplastic anemia, respectively.

The LATIN Study is being carried out in four Latin American countries: Brazil, Argentina, Colombia and Mexico. The principal hematologists and university professors in this field are taking part in it. The Advisory Board comprises well-known epidemiologists and authors of several studies on the topic, including Dr. Juan Laporte, Dr. David Kaufman and Dr. Samuel Shapiro, who provide the necessary scientific support.

While still in its initial planning phase, the LATIN Study was presented at a meeting held in the Ministry of Health entitled “Dipyrone efficacy and safety panel”.^7^ It was concluded from this meeting that withdrawing dipyrone from the market would result in increased indiscriminate use of other analgesic and antipyretic agents that are not exempt from side effects.

One of the recommendations of the panel was to follow-up the results from the LATIN Study, which would serve as a drug surveillance system. Two reports have already been sent to Anvisa (the Brazilian Health Surveillance Agency) since the panel was held; the most recent of these reports described the results from the pilot study.

## CONCLUSIONS

In conclusion, agranulocytosis is a severe disease frequently associated with the use of medicines. However, it is a rare condition and, according to data from retrospective studies^5^ and preliminary data from the pilot phase of the LATIN Study,^6^ it is even less frequent in Brazil. The data that will be available at the conclusion of the LATIN Study will enable definition of the frequency of agranulocytosis in Brazil and other Latin American countries, as well as to what extent dipyrone is associated with its onset. This information is essential for defining health policies regarding dipyrone in these countries.

## References

[1] Watts RG, Greer JP, Foerster J, Lukens JN, Rodgers GM, Paraskevas F, Glader BE (2004). Neutropenia. Wintrobe's Clinical ematology.

[2] Kaufman DW, Kelly JP, Levy M, Shapiro S (1991). The drug etiology of agranulocytosis and aplastic anemia.

[3] Kaufman DW, Kelly JP, Jurgelon JM (1996). Drugs in the aetiology of agranulocytosis and aplastic anaemia. Eur J aematol Suppl.

[4] Maluf EM, Pasquini R, Eluf JN, Kelly J, Kaufman DW (2002). Aplastic anemia in Brazil: incidence and risk factors. Am J ematol.

[5] amerschlak N, Montezuma MP, Bacal N, Szterling LN, Rosen-feld LG, Guerra CC (1993). Retrospective prevalence and incidence of drug-induced agranulocytosis in the city of São Paulo-Brazil. Rev Paul Med.

[6] amerschlak N, Maluf E, Pasquini R (2005). Incidence of aplastic Incidence of aplastic anemia and agranulocytosis in Latin America: the LATIN study. Sao Paulo Med J.

[7] Agência Nacional de Vigilância Sanitária (2001). Resolution RE no 1260, de 15 de agosto de 2001. Determina a publicação do Relatório Final do “Painel Internacional de Avaliação da Segurança da Dipirona”.

